# Team Switching in Peer-Based Learning Groups: Academic Performance and Medical Student Perspectives

**DOI:** 10.1007/s40670-025-02540-3

**Published:** 2025-11-07

**Authors:** M. Ariel Cascio, Julia Knopes, Barbara Warner, Edward McKee

**Affiliations:** 1 Center for Bioethics and Social Justice & Department of Medicine, Michigan State University, East Lansing, MI 48825 USA; 2 Department of Bioethics, School of Medicine, Case Western Reserve University, Cleveland, OH 44106 USA; 3 Psychology Department, Bowling Green State University, Bowling Green, OH 43402 USA; 4 College of Medicine, Central Michigan University, Mount Pleasant, MI 48858 USA

**Keywords:** Medical education, Team-based learning (TBL), Case-based learning (CBL), Academic outcomes, Student perspectives

## Abstract

This paper reports on academic outcomes and student perspectives on a 3-year medical education intervention study in which students were assigned to either work with the same “static” group for both team-based learning (TBL) and case-based learning (CBL) sessions, or work with a “hybrid” TBL group comprised of members from different CBL groups. Data included exam and quiz scores, surveys, and focus groups. There were no significant differences in exam scores. Differences in quiz scores were inconsistent. Survey respondents rated static groups better. Qualitative data revealed student ambivalence, which should be addressed when implementing team switching interventions.

## Background

Peer-based learning groups have proliferated in medical education in the past few decades [1, 2]. However, research has yet to study the effect that team switching has on academic outcomes and student experiences, i.e., what happens when medical students change membership to new small group teams across the pre-clerkship curriculum? This paper reports on academic outcomes and student perspectives on a medical education intervention study to address this gap, conducted at Central Michigan University College of Medicine (CMU COM). CMU COM assigned students to 8-member teams that remain together an entire academic year, across multiple courses. Courses used team-based learning (TBL) to assess and extend small group case-based learning (CBL), which is considered the pre-work for the TBL. Students also complete “Monday Morning TBLs” based on traditional pre-work with no associated CBL. The other teaching modalities used in this curriculum over this time period did not involve formalized peer interaction strategies. This format provided a unique opportunity for this intervention to potentially enhance peer-based learning by mobilizing multiple teams, one for CBL and one for TBL. This intervention expands the TBL tenet of maximizing the collective experience of the team [3] by creating hybrid TBL teams representing members from 6–7 CBL teams. This study hypothesized that the intervention would broaden the collective experience of the TBL team and strengthen the hybrid team over the static control team.

Current research does not clearly indicate how being a member of two different teams affects performance. We could find no studies comparing learning outcomes or group dynamics in medical school curricula where students switched small group membership. There has been only limited scholarship addressing the impact of changing groups between semesters in a non-medical curriculum [4]. A study on the impact of collaborative learning teams on academic performance among undergraduates found that switching groups between semesters led to reduced performance [1]. It is unclear if these findings would apply to medical students. While the same principles from group dynamics research that inform the principle of maintaining consistency throughout a course [5] might imply that consistency throughout a longitudinal curriculum is valued, other research suggests that changing group membership may allow for broader collaboration and exchange of knowledge between more cohort members [6]. Changing group membership is often advocated in non-TBL forms of cooperative learning [4, 7] and is used in business to facilitate groups learning from each other [8]. These non-academic metrics are important because peer-based learning may be instrumental not only to student learning but to professional identity formation [9].

## Activity

Data were collected over 3 years. In 2021 and 2023, the intervention ran during two courses. Each group was “static” for one and “hybrid” for the other. Changes to curriculum modality during the COVID-19 pandemic prohibited intervention in 2022; those data are included as additional “static” data points for analyses across all 3 years. Data were collected from the same two courses, held in the first 6 weeks and subsequent 7 weeks of students’ first year second semester, providing a new cohort each year.

Academic outcomes were (1) exam scores (midterm and final) for questions mapped to TBL sessions and (2) Individual Readiness Assurance Tests (IRAT), Group Readiness Assurance Tests (GRAT), and Group Application Exercises (GAE) multiple choice quiz scores from TBL sessions. These scores were compared between conditions using a two-tailed *t*-test.

The same analyses were completed for Monday Morning TBL quizzes, which have no associated CBL and in which the hybrid teams would have no experiential advantage. As the same teams were used for both TBLs, comparing results between the two might provide data that would more directly address the potential benefit of the broader experiences of the hybrid teams during the CBL pre-work. Course 1 had 17–18 case-based TBLs and 5–6 Monday Morning TBLs each year; 21–24 midterm and 35 final exam questions were mapped to case-based TBLs, 7–8 midterm and 10 final exam questions to Monday Morning TBLs. Course 2 had 20–21 case-based TBLs and 6–7 Monday Morning TBLs each year; 21–27 midterm and 45–46 final exam questions were mapped to case-based TBLs, 10–12 midterm and 21–27 final exam questions to Monday Morning TBLs.

A total of 98 survey responses were collected, comprised of answers to both closed-ended and open-ended surveys to generate both quantitative and qualitative data. After removing incomplete responses, 95 responses were included in our analyses (2021: 10 open-ended pre-survey, 4 open-ended post survey, 18 closed-ended course 1, 14 closed-ended course 2; 2022: 5 open-ended pre-survey, 8 open-ended post survey, 9 closed-ended course 1, 10 closed-ended course 2; 2023: 1 open-ended pre-survey, 6 open-ended post survey, 2 closed-ended course 1, 8 closed-ended course 2). Closed-ended questions were adapted from previous scales of TBL group functioning [10–12] in domains of overall assessment (e.g., “My TBL group has worked well together”), clinical reasoning (e.g., “The dialogue and discussion on my TBL group helped me learn about decision making”), professional development (e.g., “I have found that working with my TBL group helps me develop skills in working with others”), and knowledge (e.g., “I am comfortable teaching another student on my current TBL group when they do not know something”). The research team developed questions to assess switching preference, detailed in “Results and Discussion”.

Students (*n* = 7) completed focus groups in 2021 and 2023, which were transcribed verbatim and thematically coded by the research team using a defined set of themes and valence (positive, negative, neutral) of participants’ stance. Themes included collaboration, learning resources, studying, and remote learning. The codebook, created specifically for this study, operationalized each theme.

The CMU Institutional Review Board determined that this project did not meet the definition of human subject research under the purview of the IRB. Students provided consent for surveys and focus groups. Assessment scores were deidentified.

## Results and Discussion

Overall, quantitative data revealed no impact of the team switching intervention on academic outcomes including quiz scores, though qualitative data indicated that students were highly ambivalent about team switching and its impact on their learning. There were no significant differences in exam scores mapped to CBL/TBL between static and hybrid groups in either course in any year or across all 3 years. Comparing Monday Morning TBLs, there were some significant differences in course 1 when considering all 3 years combined, with hybrid performing significantly better on the midterm (static mean = 0.7927 ± 0.15329, hybrid mean = 0.8306 ± 0.14953; *p* = 0.031) and static on the final (static mean = 0.7959 ± 0.13955, hybrid mean = 0.7653 ± 0.12431; *p* = 0.048). Differences in means were minor. There were no significant differences in course 2 and no significant differences in 2021 and 2023 only (Table 1).

**Table 1:** Exam scores. **Bold**, *t*-test two-sided *p*-value < 0.05

			Static mean ± SD	Hybrid mean ± SD	*t*	df	*t*-test two-sided *p*-value
Overall	Course 1 midterm	Case-based TBL	0.8412 ± 0.081	0.86 ± 0.088	−1.959	318	0.051
		**Monday Morning TBL**	**0.7927 ± 0.153**	**0.831 ± 0.15**	**−2.164**	**318**	**0.031**
	Course 1 final	Case-based TBL	0.8608 ± 0.082	0.878 ± 0.071	**−**1.933	316	0.054
		**Monday Morning TBL**	**0.7959 ± 0.14**	**0.765 ± 0.124**	**1.981**	**316**	**0.048**
	Course 2 midterm	Case-based TBL	0.8162 ± 0.105	0.83 ± 0.095	**−**1.077	317	0.282
		Monday Morning TBL	0.8315 ± 0.129	0.811 ± 0.141	1.287	317	0.199
	Course 2 final	Case-based TBL	0.8233 ± 0.086	0.824 ± 0.079	**−**0.027	317	0.979
		Monday Morning TBL	0.819 ± 0.102	0.841 ± 0.099	**−**1.79	317	0.074

There were statistically significant differences in TBL scores between hybrid and static teams in some analyses. Across all years, in course 1, hybrid groups performed significantly better on IRAT; in course 2, static groups performed significantly better in GAE; the same groups performed significantly better in GRAT regardless of hybrid or static condition. In course 2, hybrid groups scored significantly higher in GRAT in 2021, and static groups scored significantly higher in GAE in 2023. Within Monday Morning TBLs, which do not rely on CBL pre-work, there were several significant (*p* < 0.05) differences. Hybrid groups performed significantly better in course 1 2023 GAE, Overall IRAT, Overall GAE; and 2021 Course 2 GRAT, Overall IRAT, and Overall GRAT. Static groups performed significantly better in 2021 course 1 GRAT and Overall GRAT. Differences in means ranged from 0.02 to 0.11 (Table 2). These data do not suggest that team switching has a consistent positive or negative effect on students’ overall performance. Results of a lowest-quartile analysis, like those just discussed, were mixed and do not contribute to the overall discussion. These results are available upon request.

**Table 2:** Quiz scores. **Bold**, *t*-test two-sided *p*-value < 0.05. *Italic*, Levene’s test *p*-value < 0.05, equal variances not assumed

				Static mean ± SD	Hybrid mean ± SD	*t*	df	*t*-test two-sided *p*-value
2021	Course 1	Case-based TBL	IRAT	0.7574 ± 0.098	0.7543 ± 0.10731	0.15	101	0.881
			GRAT	0.9796 ± 0.018	0.972 ± 0.02825	1.569	101	0.12
			*GAE*	*0.6903 ± 0.081*	*0.6731 ± 0.06165*	*1.193*	*82.69*	*0.236*
		Monday Morning TBL	IRAT	0.6393 ± 0.128	0.665 ± 0.13391	**−**0.989	101	0.325
			***GRAT***	***0.9895 ± 0.038***	***0.949 ± 0.06024***	***4.17***	***95.384***	***0.000***
			GAE	0.7391 ± 0.07629	0.7566 ± 0.06289	**−**1.272	101	0.206
	Course 2	Case-based TBL	IRAT	0.7205 ± 0.114	0.725 ± 0.1161	0.199	103	0.842
			***GRAT***	***0.8893 ± 0.125***	***0.9444 ± 0.01807***	***3.354***	***61.119***	***0.001***
			GAE	0.6982 ± 0.114	0.6921 ± 0.06673	**−**0.32	103	0.75
		Monday Morning TBL	IRAT	0.6225 ± 0.122	0.6256 ± 0.16378	0.111	103	0.912
			***GRAT***	***0.9169 ± 0.139***	***0.9783 ± 0.01924***	***3.34***	***60.827***	***0.001***
			GAE	0.7385 ± 0.107	0.7454 ± 0.06753	0.38	103	0.705
2023	Course 1	Case-based TBL	IRAT	0.7601 ± 0.063	0.7598 ± 0.06409	0.016	104	0.987
			GRAT	0.9191 ± 0.021	0.9198 ± 0.01956	**−**0.06	11	0.954
			GAE	0.8615 ± 0.049	0.8626 ± 0.03747	**−**0.134	104	0.894
		Monday Morning TBL	IRAT	0.6395 ± 0.111	0.6693 ± 0.1192	**−**1.321	104	0.189
			GRAT	0.8571 ± 0.056	0.8545 ± 0.04068	0.096	11	0.925
			***GAE***	***0.7431 ± 0.079***	***0.779 ± 0.03548***	**−*2.923***	***62.617***	***0.005***
	Course 2	Case-based TBL	IRAT	0.6681 ± 0.086	0.6664 ± 0.09951	0.096	105	0.924
			*GRAT*	*0.9766 ± 0.032*	*0.9646 ± 0.04061*	*1.649*	*86.388*	*0.103*
			***GAE***	***0.8359 ± 0.042***	***0.8128 ± 0.03649***	***3.024***	***104.033***	***0.003***
		Monday Morning TBL	IRAT	0.557 ± 0.122	0.5688 ± 0.13541	**−**0.474	105	0.637
			GRAT	0.9273 ± 0.115	0.8974 ± 0.13028	1.257	105	0.211
			GAE	0.891 ± 0.09	0.8518 ± 0.12205	1.913	105	0.058
Overall	Course 1	Case-based TBL	**IRAT**	**0.7292 ± 0.094**	**0.7571 ± 0.08785**	**−2.583**	**309**	**0.01**
			***GRAT***	***0.9616 ± 0.031***	***0.9449 ± 0.03616***	***4.126***	***211.399***	** < *0.001***
			*GAE*	*0.7796 ± 0.081*	*0.7687 ± 0.10785*	*0.943*	*189.703*	*0.347*
		Monday Morning TBL	**IRAT**	**0.6861 ± 0.158**	**0.7775 ± 0.15162**	**−5.004**	**309**	** < 0.001**
			**GRAT**	**0.8945 ± 0.158**	**0.8079 ± 0.16919**	**4.534**	**309**	** < 0.001**
			**GAE**	**0.7312 ± 0.068**	**0.7694 ± 0.0626**	**−4.931**	**309**	** < 0.001**
	Course 2	Case-based TBL	IRAT	0.6828 ± 0.1	0.6954 ± 0.11142	**−**0.985	312	0.325
			***GRAT***	***0.8444 ± 0.113***	***0.9318 ± 0.0218***	**−*11.07***	***255.793***	** < *0.001***
			**GAE**	**.7332 ± 0.06235**	**0.7679 ± 0.05196**	**−5.028**	**309**	** < 0.001**
		Monday Morning TBL	IRAT	0.5514 ± 0.141	0.5969 ± 0.15201	**−**2.55	312	0.011
			***GRAT***	***0.8071 ± 0.134***	***0.9151 ± 0.06899***	**−*9.408***	***302.01***	** < *0.001***
			GAE	0.7939 ± 0.119	0.7992 ± 0.11198	**−**0.363	312	0.717

**Fig. 1:**
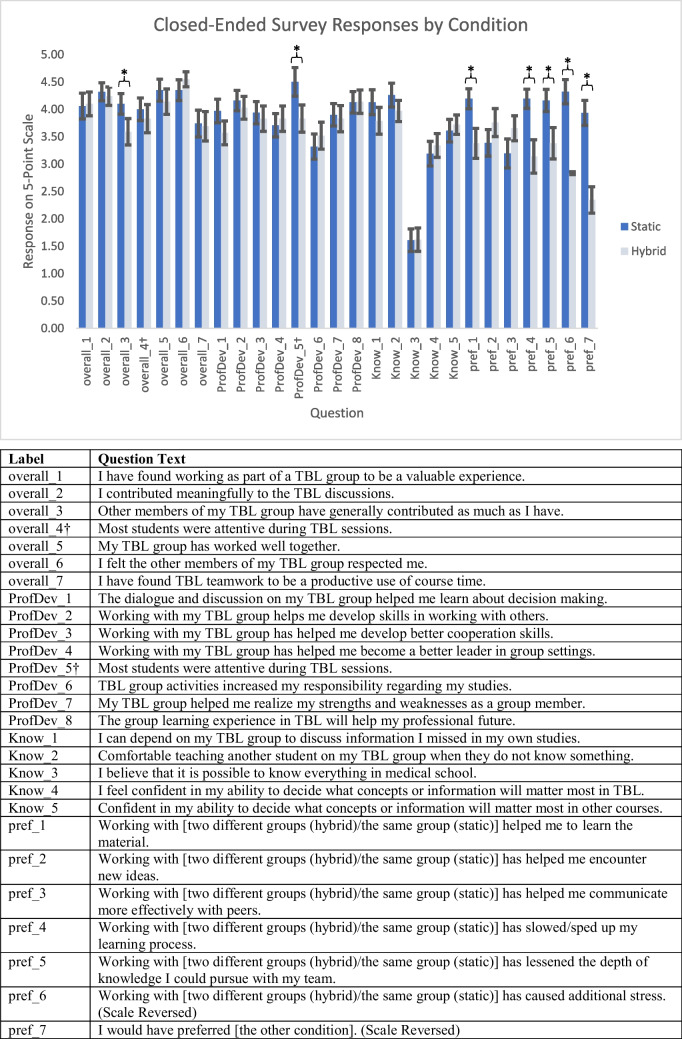
Survey responses. Average responses on a 5-point scale. Bars indicate standard error of the mean. Asterisks (*) indicate that error bars are not overlapping. Daggers (†) indicate this question was inadvertently duplicated years 1 and 3 (42 respondents). As responses differed, both are reported here. Non-overlapping error bars suggest difference between respondents in static and hybrid groups. Questions presented in the order they were asked

Survey respondents rated both conditions fairly high in most questions across all domains. The largest differences were in questions about switching preferences. Respondents in the static group indicated a preference for the hybrid condition less often than respondents in the hybrid condition indicated a preference for static (Fig. 1).

Qualitative results, published at length elsewhere [13, 14], demonstrated mixed sentiments. In focus groups and open-ended surveys, students expressed concern about streamlining their studies to learn large amounts of material efficiently [13]. Switching teams could impede this, as one student shared there was too little time to become “comfortable” with new members and they never “reach[ed] the level” of “being able to efficiently move through a case” (Focus Group Transcript 6/24/2021). Another student admitted that “awkwardness from working with new people impeded discussion” (Open-ended Survey 2021). However, other students have recognized the value of learning from peers with different academic strengths and educational backgrounds [14]. A participant reported that “everyone had a diverse background that helped with our understanding of many topics” in their small group.

Given that there were few overall differences in academic outcome measures, it is possible that the anticipated advantages of maximizing broader CBL experiences are matched by the anticipated disadvantages of needing to form and norm a new team. It is also possible that the CBL experience for each team is similar enough that hybrid teams did not draw on greater breadth.

## Conclusion

Because multiple peer-based learning groups do not consistently impact academic outcomes, educators could pursue either condition based on their mission and goals. Students recognize advantages and disadvantages of each condition. Educators using hybrid groups should address student concerns regarding increased stress and negative impacts on perception of learning, while sharing with students the benefits of learning from peers who bring unique expertise into small group settings.

## Data Availability

The data that support the findings of this study are not openly available due to reasons of sensitivity and are available from the corresponding author upon reasonable request.
